# The Dapsone Hypersensitivity Syndrome revisited: a potentially fatal multisystem disorder with prominent hepatopulmonary manifestations

**DOI:** 10.1186/1745-6673-1-9

**Published:** 2006-06-06

**Authors:** Semaan G Kosseifi, Bhuvana Guha, Dima N Nassour, David S Chi, Guha Krishnaswamy

**Affiliations:** 1 Department of Internal Medicine, Quillen College of Medicine, East Tennessee State University, Johnson City, TN, USA; 2 Department of Internal Medicine, Division of Allergy and Clinical Immunology, Quillen College of Medicine, East Tennessee State University, Johnson City, TN, USA

## Abstract

4,4'-Diaminodiphenylsulphone (Dapsone) is widely used for a variety of infectious, immune and hypersensitivity disorders, with indications ranging from Hansen's disease, inflammatory disease and insect bites, all of which may be seen as manifestations in certain occupational diseases. However, the use of dapsone may be associated with a plethora of adverse effects, some of which may involve the pulmonary parenchyma. Methemoglobinemia with resultant cyanosis, bone marrow aplasia and/or hemolytic anemia, peripheral neuropathy and the potentially fatal dapsone hypersensitivity syndrome **(DHS)**, the focus of this review, may all occur individually or in combination. **DHS **typically presents with a triad of fever, skin eruption, and internal organ (lung, liver, neurological and other systems) involvement, occurring several weeks to as late as 6 months after the initial administration of the drug. In this sense, it may resemble a **DRESS syndrome **(Drug Rash with Eosinophilia and Systemic Symptoms). **DHS **must be promptly identified, as untreated, the disorder could be fatal. Moreover, the pulmonary/systemic manifestations may be mistaken for other disorders. Eosinophilic infiltrates, pneumonitis, pleural effusions and interstitial lung disease may be seen. This syndrome is best approached with the immediate discontinuation of the offending drug and prompt administration of oral or intravenous glucocorticoids. An immunological-inflammatory basis of the syndrome can be envisaged, based on the pathological picture and excellent response to antiinflammatory therapy. Since dapsone is used for various indications, physicians from all specialties may encounter **DHS **and need to familiarize themselves with the salient features about the syndrome and its management.

## Background

Dapsone has been used for many indications because of its antibiotic and anti-inflammatory effects [1]. Not only has it been the drug of choice for the treatment of leprosy (Hansen's disease) since the middle of the 20^th ^century, but it has also been used for the treatment of brown recluse spider bite [1,2], dermatitis herpetiformis, vesicobullous dermatoses, cutaneous vasculitis, polyarteritis nodosa, nodulocystic acne, cutaneous mycetoma and multiples other dermatologic indications all of which may be seen as manifestations in certain occupational diseases [1]. Since the advent of the era of the Acquired Immunodeficiency Syndrome (AIDS), dapsone has been increasingly utilized in the chemoprophylaxis of *Pneumocystis carinii *infection in combination with Trimethoprim/Sulfamethoxazole. This has led to increasing incidence of dapsone-related complications. Table 1 lists the multiple adverse effects of Dapsone, including the dapsone hypersensitivity syndrome **(DHS) **and **DRESS **syndrome. Hemolysis (more likely to occur with deficiency of glucose 6-phosphate dehydrogenase or G6-PD), bone marrow aplasia, renal disease, peripheral neuropathy, methemoglobinemia, nausea, dizziness, fatigue and other systemic manifestations may occur singly or in combination in patients on dapsone therapy. Of these, **DHS **is characterized by the onset of fever, skin eruption and internal organ involvement several weeks to as late as 6 months after patients are given this drug. Untreated the syndrome can lead to severe organ dysfunction and even death. The definitive mechanism for **DHS **is unclear, but it is hypothesized that it is mediated by immune activation and elaboration of inflammatory cytokines. This case report emphasizes the pulmonary effects associated with **DHS**. The following sections provide an overview of the presentations, pathogenesis, diagnosis and management of **DHS**.

**Table 1:** Adverse reactions to Dapsone

System	Manifestations	Mechanisms
**Systemic**		
• *DHS	Dermatitis, hepatitis	Idisosyncratic
• **DRESS syndrome	Dermatitis, eosinophilia	Idiosyncratic
• Nonspecific	Nausea, headache, dizzy Weakness/fatigue	Predictable
**Hematological**	Hemolytic anemia	Predictable (G6PDD)
	Methemoglobinemia	Predictable
**Neurological**	Peripheral neuropathy	Predictable
**Dermatological**	Stevens-Johnson Syndrome	Idiosyncratic
	Toxic epidermal necrolysis	Idiosyncratic
**Hepatic**	Colestasis, hepatitis	Idiosyncratic
**Renal**	Nephritis	Idiosyncratic
**Pulmonary**	Pneumonitis, ***PIE	Idiosyncratic
**Thyroid**	Hypothyroidism	Idiosyncratic

DHS = dapsone hypersensitivity syndrome, DRESS = drug rash, eosinophilia and systemic symptoms, PIE = pulmonary infiltration with eosinophilia

### Case report

A twenty-one-year old previously healthy nursing student, with no significant past medical or surgical history, presented with an apparent reaction to an insect bite on her left shin. She was evaluated and based on the presumed diagnosis that this represented a hypersensitivity reaction to an insect bite, and she was treated with dapsone (100 mg twice a day for 7 days) as an anti-inflammatory agent. This was based on some data supporting the use of this drug for insect bites [2], although this now appears to be a controversial approach. After seven days of therapy, the patient developed progression of her symptoms, developing fever with chills, myalgia and nausea associated with diffuse abdominal pain, dark-colored urine and jaundice. She also noticed a new onset non-pruritic, ascending, maculo-papular skin eruption, with progressive shortness of breath. She denied cough, hemoptysis, or chest pain at this time. On admission, the patient was jaundiced, tachypenic with a respiratory rate of 28 breath/minute, heart rate of 103 beats/minute, blood pressure of 130/92 mm of Hg, and a temperature of 99°F. The patient's oxygen saturation was 86 % on room air, and subsequently she was given higher amount of oxygen, to the point that she was requiring 100 % non-rebreather mask in order to maintain her oxygen saturation above 92 %. Oral mucosa was normal, with no visible lesions. Neck examination was supple, with no palpable lymphadenopathy or evidence of thyroid enlargement. Lung examination revealed bilateral diffuse crackles, decreased breath sounds in both bases and dullness to percussion. Cardiac examination showed tachycardia without audible murmur while the abdominal examination revealed hypoactive bowel sounds with diffuse mild tenderness on deep palpation, without palpable organomegaly or evidence of rebound tenderness. The patient demonstrated a diffuse maculopapular eruption with sparing of the hands, feet, and mucosa. There was no blanching on pressure. There was a healing ulcer on her left shin with a dried-up scar (site of presumed insect bite), without surrounding erythema or other signs of inflammation or infection. The absence of significant necrosis or surrounding inflammation suggested that the symptoms the patient was experiencing were independent of the insect bite.

**Figure 1:**
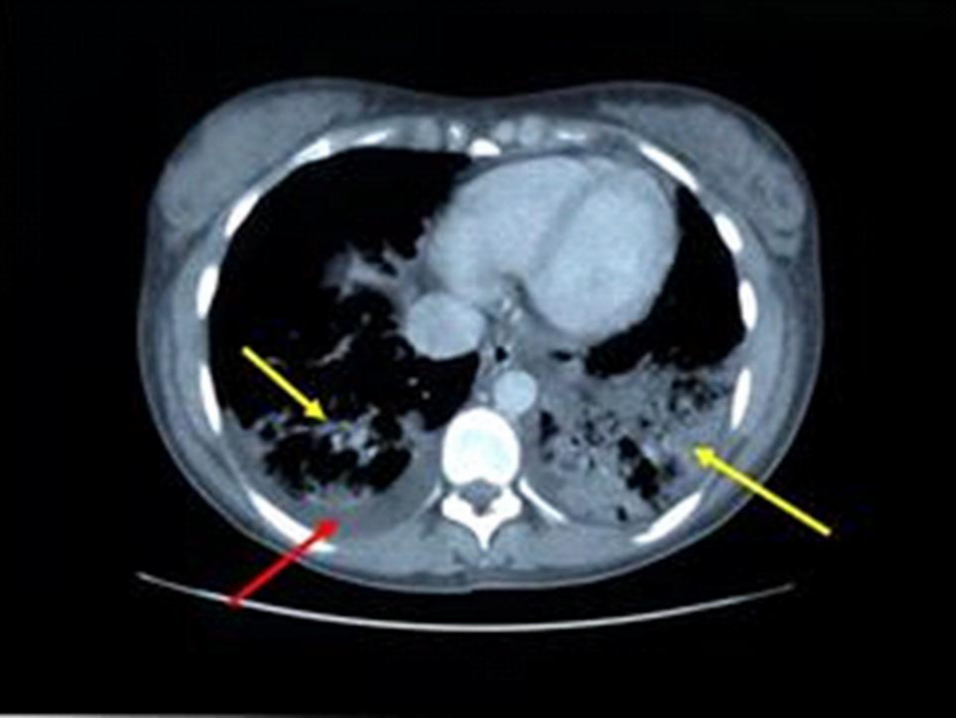
Computed chest tomography of the patient described in this report, showing bilateral interstitial infiltrates (yellow arrow) and pleural effusions (red arrow). Image taken at a mid-thoracic level.

The admitting laboratory data is provided in Table 2. Liver function abnormalities consistent with transaminitis were seen. The peripheral smear review showed a normocytic, normochromic anemia with mild neutrophilia and toxic granulations. The patient also demonstrated mild anisocytosis, poikilocytosis and reticulocytosis. The urine sample tested positive for blood but the patient had negative urine, blood and stool cultures. A chest roentgenogram was done on admission and showed a diffuse bilateral air space disease involving the majority of the lung with sparing of the lung apices with bilateral pleural effusions. Computed tomography of the abdomen and pelvis with contrast showed gallbladder wall-thickening and mild dilatation, with small amount of abdominal fluid (ascites). Computed tomography of the chest showed bilateral infiltrates mainly on the left, along with bilateral pleural effusion (Figure 1). Evaluation for other infectious and/or immunological etiologies was negative, and included a hepatitis panel, ELISA for HIV 1–2 and testing for Legionella, Rocky Mountain Spotted Fever, cold agglutinins, Group B streptococcus, Influenza A and B, Monotest, direct Coombs, Leptospirosis, Ehlerichiosis, Ebstein-Barr virus (EBV), Cytomegalovirus (CMV) and Parvovirus.

**Table 2:** Representing the laboratory data.


**Measurement**	**admission**	**day 1**	**day 2**	**day 3**	**day 4**	**day 5**	**Normal Values**
WBC	8	8.3	10.5*	6.7	7.3	8.3	4.0 – 9.2 × 10^3^/mm3
Hb	6.9*	8.6*	11.5*	11.9*	13.3	15	12.1 – 15.2 g/dL
Platelet	217	246	318	380	468*	425	150 – 450 × 10^3^/mm^3^
ALT	103*	100*	86*	100*	130*	77*	10 – 60 IU/L
AST	179*	111*	91*	75*	68*	30	10 – 42 IU/L
ESR		21*	11				0 – 20 mm/hour
T.Bil.	8*	5*	1.8*	2.3*	1.5*	1.2*	0.2 – 1 mg/dL
LDH		778*		1182*	589*		90 – 180 IU/L
CPK	112						30 – 165 IU/L

*means abnormal values.

Black arrow indicating the timing when intravenous glucocorticoids were given to the patient.

WBC = White blood count, MCV = Mean corpuscular volume, Hb = Hemoglobin, ALT = Alanine aminotransferase, AST = Aspartate aminotransferase, T.Bil = Total bilirubin, LDH = Lactate dehydrogenase, INR = International ratio, CPK = Creatinine phosphokinase.

Based on the clinical picture of multisystem involvement, lack of a defining microbiological cause and the progression of disease, a diagnosis of DHS was made.

The patient was immediately initiated on intravenous glucocorticoids (methylprednisolone) with dramatic improvement in her symptoms and clinical disease. This improvement is shown in Table 2 and includes dramatic reversal of anemia, improvement in transaminitis and in inflammatory parameters. This was also evidenced by stabilization of hypoxemia with less need for oxygen supplement.

### Dapsone Hypersensitivity Syndrome

#### Background

**DHS **is characterized by a hypersensitivity response to the drug, dapsone which is a sulfone. Dapsone (4,4'-Diaminodiphenylsulphone) is used mainly as an anti-inflammatory and anti-bacterial agent for the treatment of skin diseases, bacteria, and fungi [1]. The anti-inflammatory effects of dapsone are mainly related to its interference with neutrophil chemotactic migration and adherence [1]. Other multiple mechanisms are listed in the literature. These describe suppression of neutrophil recruitment, inhibition of local production of toxic respiratory and secretory products, as well as formation of oxidants. These not only kill bacteria but also damage bystander tissues. In addition, dapsone can inhibit the release of prostaglandins and leukotrienes by blocking their inflammatory effects [1,3-5]. Overall, the side effects of dapsone are very low if plasma concentration is below 5 mg/l [1,4].

A list of adverse effects of dapsone (some predictable, some idiosyncratic or allergic) is provided in Table 1.

A rare syndrome, **DHS **which was first described by *Allday, Lowe*, and *Barnes *[4,5] as a hypersensitivity vasculitis syndrome [1,3-8]. The incidence of **DHS **ranges from 0.5% to 3 % [1,3,6,8], while the median latency before symptoms onset can be as early as 2 to 6 hours in previously sensitized patients, to as late as 6 months. This variability of the latency time was explained by several factors. Some of those were related not only to the variability of the acetylators but also to the dose and the modality of treatment [1,4,6,8]. This wide variability of the latency period suggests a multi-organ hypersensitivity reaction, but the exact mechanisms are unclear and reviewed later [4-7].

### Clinical features

The classic triad of **DHS **consists of fever, eruption, and internal organ involvement (Table 3). Fever, hepatitis, exfoliative dermatitis, adenopathy and hemolytic anemia might be seen in varying combinations and sequences [9]. While traditionally a hepatitis or transaminitis is seen, cholangitis has also been described as a component of the **DHS **[9]. Studies have shown that this syndrome may begin as early as 7–10 days after administration of the drug or as late as 6 months into therapy with dapsone. Cutaneous lesions can range from erythematous papules as in our patient, to plaques, pustules, and eczematous lesions [1,3-8]. The severity of the cutaneous changes does not correlate with the severity or extent of internal organ involvement which may remain asymptomatic of even life-threatening [10]. Cutaneous lesions usually begin to resolve 2 weeks after stopping therapy. Some patients may also develop severe dermatitis and complications, such as the Steven Johnson Syndrome or toxic epidermal necrolysis (Table 1). These patients may experience prolonged morbidity and even mortality with the complications. Especially in severe cases, malnutrition, protein loss and secondary infection may complicate the illness, and these patients need to be monitored for complications more aggressively. In traditional **DHS**, antibiotics have little or no role unless obvious infection, cellulitis or sepsis is present.

**Table 3:** DHS clinical manifestations.

**Systemic**
1. Fever*
2. Pneumonitis*
3. Lymphadenopathy
4. Hepatitis*
5. Hemolytic anemia*
6. Carditis
**Dermatological**
1. Exfoliative dermatitis
2. Eczematous/maculopapular eruption*
3. Oral erosions
4. Vesicles and bullae
5. Photosensitivity
**Laboratory**
1. Hemolysis*
2. Anemia*
3. Eosinophilia
4. Atypical lymphocytosis
5. Transaminitis/elevated bilirubin/alkaline phosphatase*
6. Hypogammaglobulinemia

*-manifestations present in the patient described.

### Pulmonary manifestations

Infiltrative lung disease is the most common pattern of drug-induced injury [10]. This pattern can cause interstitial lung damage, alveolar damage or vasculitic involvement (e.g., vasculitis) [10]. As listed in Table 3, **DHS **can also involve the lung, with 10 cases reported in the literature. In the patient described by us, pulmonary infiltrates and pleural effusions with severe hypoxemia were present suggesting interference with alveolocapillary oxygen transfer. Other manifestations have also been described in the literature.

Table 4 summarizes the different pulmonary manifestations of dapsone. Pulmonary manifestations of the **DHS **are sometimes dominant features, as occurred in our patient. Manifestations have included the development of eosinophilic pneumonia [11-15], hypersensitivity pneumonia [16], pleural effusion [17]. Many reported cases of eosinophilic pneumonia have an associated systemic blood eosinophilia, which however, was not present in our case. The clinical/radiological presentation of our patient, along with the dramatic clinical response to glucocorticoids, strongly suggest a drug-induced hypersensitivity pneumonitis. The rapid clinical deterioration seen with **DHS **can lead to respiratory failure as seen in our patient and sometimes death, if untreated or unrecognized.

**Table 4:** Reported cases of Dapsone Hypersensitivity Syndrome with pulmonary manifestations.

**Age/Sex**	**Pulmonary manifestations**	**Treatment**	**Reference**
22/M'	Crepitations	DW*, Steroid	(6)
47/F	Pulmonary eosinophilia	DW, Steroid	(11)
65/F	Pulmonary eosinophilia	DW	(12)
45/M	Pulmonary eosinophilia	DW, Steroid	(13)
60/F	Eosinophilic pneumonia	DW	(14)
23/F	Eosinophilic pneumonia	DW, Steroid	(15)
31/M	Eosinophilic pneumonia with pulmonary infarction	DW, Steroid	(15)
37/F	Eosinophilic pneumonia	DW	(15)
40/F"	Hypersensitivity pneumonitis	DW, Steroid	(16)
15/M	Right sided pleural effusion	DW, Steroid	(17)

*DW means drug withdrawal with supportive therapy only.

' M = male, " F = female.

### Other manifestations

Other manifestations of this syndrome include hepato-biliary dysfunction (such as jaundice[1], hepatomegaly [1]and cholangitis [9], splenomegaly[1], eosinophilia[1,13], photosensitivity[1], elevated sedimentation rate [1], and a mononucleosis picture that can mimic EBV and CMV infection [1,7]. In addition, **DHS **can include peripheral neuropathy [18], psychosis [19], renal involvement (such as nephrotic syndrome [1] and renal papillary necrosis [20] and pancreatitis [17] as found in our patient. As listed in Table 3, a maculopapular eruption, bullous disease, photosensitivity and oral erosions can occur.

### Differential diagnosis

The differential diagnosis of multisystem illness presenting in a patient on dapsone is shown in Table 5. These include diseases such as: **DRESS **syndrome and its variants, vasculitis (Churg Strauss syndrome), Hypereosinophilic syndrome, **TENS **(Toxic epidermal necrolysis syndrome), Steven Johnson Syndrome, Still's disease, Hematological disorders (leukemia, lymphoma), paraneoplastic disorders and certain connective tissue disorders. We will discuss a few of these important conditions in this section.

**Table 5:** Differential Diagnosis of DHS

DRESS syndrome and it's variants
Vasculitis (Churg Strauss syndrome)
Hypereosinophilic syndrome
Toxic epidermal necrolysis
Steven Johnson Syndrome
Still's disease
Hematological disorders (leukemia, lymphoma)
Paraneoplastic disorders
Certain connective tissue disorders

Systemic organ involvement is often a feature of both **DHS **and the **DRESS **syndrome. In the case of the latter, drug rash, eosinophilia and systemic symptoms are often present. **DRESS **syndrome can be seen with a variety of medications, including anticonvulsants, sulfonamides, allopurinol, calcium channel blockers, NSAIDS (Non-steroidal anti-inflammatory drugs) and dapsone [21]. Fever, skin eruption, adenopathy, eosinophilia and internal organ involvement might also be seen [21]. Slow acetylators may be associated with an increased risk for development of **DRESS **syndrome. **DHS **can be considered a variant of the **DRESS **syndrome.

**TENS **and Stevens-Johnson syndrome represent a dermatologic emergency. They are characterized by diffuse erythematous or purpuric macules with involvement of more than 30 % of body surface area with epidermal necrosis and mucosal membranes involvement. It is important to note, that both disorders may overlap. A prodromal phase, similar to a flu-like illness, lasting up to14 days, may precede the skin eruption. The acute phase of **TENS **consists of persistent fevers, generalized epidermal sloughing and mucous membrane involvement [22].

Cutaneous vasculitis which can be part of a systemic disease needs also to be excluded. The evaluation of such patients needs a complete work-up for the presence of systemic disease. Skin biopsy and immunofluorescence studies may also helpful in the diagnosis of cutaneous vasculitis in which immunoglobulin and complement deposition are found [23].

If dermatitis and pulmonary disease is the dominant feature, necrotizing vasculitides and Churg Strauss syndrome needs to be excluded. If dermatological manifestations dominate the presentation, with or without mucosal involvement, Steven Johnson Syndrome and **TENS **need to be excluded. In some patients with a dominant eosinophilia and pulmonary infiltration, **PIE **syndrome [10] needs to be excluded.

### Evaluation

Laboratory tests can include a complete blood count and differential, comprehensive chemistry profile, sedimentation rate, urine analysis, arterial blood gases and a chest roentgenogram. In selected cases, chest computed tomography, hepatic ultrasound and/or liver or skin biopsy may be required. Skin biopsy may not be specific but will assist in excluding vasculitis or hematological malignancies. It might be important to obtain a thyroid stimulating hormone level in patients 3–4 months after the diagnosis of **DHS **as detailed below.

### Molecular and immunopathogenesis of DHS

The exact immune mechanism behind **DHS **is unclear. A few mechanisms have been proposed. For one, **DHS **might be a combination of type I, type IV, and perhaps type III Gel and Coombs hypersensitivity reactions [5]. Alternately, **DHS **could be a modified graft versus host disease mediated by activated T-lymphocytes [5]. It is worth noting that **DHS **is not a dose-related effect [3-6,8], whereas dapsone hepatotoxicity is a dose-dependent effect [3].

According to *Prussick and Shear*, there is some evidence suggesting that the metabolic differences in the production and detoxification of reactive metabolites are an important factor in sulfonamide hypersensitivity reactions [24]. After absorption from the gastrointestinal tract, dapsone is transported through the portal circulation to the liver where it is metabolized primarily via two pathways: N-acetylation and N-hydroxylation [1,24]. N-acetylation which has a bimodal pattern of activity (slow and fast acetylation), has been shown not to determine total clearance of dapsone [24]. However, the N-hydroxylation pathway which is mediated primarily by human liver microsomal enzymes P4503A4, 2C6, and 2C11 [1,24], is shown to be the initial step in the formation of toxic intermediate metabolites, such as nitrosamines and possibly other compounds, which can induce hemolytic anemia and methemoglobinemia [24]. It is presumed that these molecules are also important in the pathogenesis of **DHS**. While N-hydroxylation yields a potentially toxic metabolite known as the hydroxylamine, produced by cytochrome P-450, acetylation by N-acetyltransferase yields the nontoxic metabolites monoacetyl dapsone and diacetyl dapsone [1]. Moreover, it has been shown that a reduction in either quantity or activity of N-hydroxylation enzyme systems resulted in decreased total clearance of dapsone. Furthermore, this information is supported by studies showing an extensive population and individual variation in this ability involving both genetic (increased or decreased P450 activity, decreased reduced glutathione [GSH]) and environmental (drugs or chemicals such as smoking inducing P450, cirrhosis and drugs inhibiting P450, decreased GSH such as in AIDS, deficiency of antioxidants such as Vitamin E, C, selenium) [24]. Fortunately, other factors such as increased age and preexisting liver disease (e.g., cirrhosis) offer relative protection against adverse events because of decreased enzyme activity and, therefore, decreased production of toxic metabolites [24]. However, with the lack of clinical studies, those aforementioned protective factors remain speculations.

The mechanisms behind the pulmonary manifestations may be similar, and due to the occurrence of eosinophilia and pulmonary infiltrations, suggest the role for cytokines such as tumor necrosis factor alpha (TNF-α), interleukin-5 (IL-5) and chemokines [25,26]. These have not been routinely studied. The rapid response to glucocorticoids [27] also suggests that activation and nuclear translocation of nuclear factor kappaB (NF-kappa B) may occur, resulting in a massive inflammatory response [25,26,28]. Glucocorticoids have also been shown to have both genomic and nongenomic mechanisms that influence inflammatory diseases [27]. How such events may relate to the ultimate pathogenesis and evolution of this syndrome are unclear but need further examination.

### Treatment

Treatment options for **DHS **are listed in Table 6. The main treatment for **DHS **is immediate discontinuation of the drug with initiation of oral or parenteral glucocorticoids, depending on severity [1,5]. Glucocorticoids (such as prednisone, prednisolone or methylprednisolone) have profound anti-inflammatory actions, as summarized earlier. The usual starting doses are mentioned in Table 6, but are approximations only. Glucocorticoids should be tapered gradually over a period of more than one month because dapsone is found to persist in the body for up to 35 days [5]. It should be remembered that glucocorticoids are medications with multiple side effects, including hyperglycemia, hypokalemia, osteoporosis, glaucoma and cataracts. Patients with risk factors for any of these complications need close monitoring. Measurements of blood sugar, bone mineral density, frequent measurements of electrolytes and a thorough ophthalmologic evaluation all constitute appropriate monitoring strategies.

**Table 6:** Treatment approaches to DHS

Intervention	Comments
1. Withdrawal of offending medication (dapsone)	Drug discontinuation
2. Supportive therapy	
• Volume replacement	Intravenous fluid replacement
• Nutritional support	Enteral or parenteral nutrition
• Antibiotics	Early antibiotic institution in case of concomitant sepsis
• Skin care	Preventing skin superinfection
3. Specific therapy	
• Glucocorticoids	Recommended dose 1 mg/kg/day
• Thyroid hormone replacement	Associated late hypothyroidism
• Family counseling	Genetic factors involvement

According to *Reeve et al*., [8] patients can be desensitized to dapsone following **DHS**, by a very gradual re-introduction of the drug at low doses [8], but this hypothesis was not supported by *Pavithran K. et al*. [4] who did not advise re-challenge with dapsone. According to the literature, patients with viral hepatitis (B, E) are at increased risk for the development of **DHS**, suggesting the need to perform a screening test for hepatitis B before starting dapsone [5,29].

Another important issue to remember in the management of patients with **DHS **is that those patients might be at higher risk for the development of hypothyroidism after three months, suggesting the need to repeat thyroid function tests at three-month intervals and considering thyroid replacement therapy if the patient develops clinical hypothyroidism as a delayed complication (Table 6). The etiology attributed to the development of hypothyroidism, seems linked to the presence of autoantibodies, including antimicrosomal antibodies [30]. In fact, patients who are unable to detoxify reactive metabolites produced by thyroid peroxidase will be more in favor of developing a hypersensitivity reaction leading to hypothyroidism [30].

It is important to consider supportive therapies in patients with more severe and protracted illness as listed in Table 6. Nutritional support (either total parenteral nutrition, nutrition supplementation or enteral feeding), meticulous fluid and electrolyte balance, control and prevention of infectious complications (cellulitis, sepsis) and skin care if necrotizing disease (**TENS **or Steven Johnson Syndrome were to ensue). For patients with dapsone-induced hemolysis, Vitamin E supplementation might be beneficial while in patients with methemoglobinemia coadministration of cimetidine can have an ameliorative effect.

In selected cases where glucocorticoids cannot be used or are associated with severe complications (glaucoma, severe osteoporosis, hyperglycemia or severe psychosis), alternative drug therapies might be required. Unfortunately, this being a rare disorder, other therapeutic options such as methotrexate, azathioprine, cyclosporine or hydroxychloroquin, have not been vigorously studied. These drugs may provide benefit in the occasional patient.

Severe internal organ involvement such as carditis, hepatitis, pneumonitis and colitis, can cause death. These can occur at any time and hence vigilance is required in these sick patients. Also to be remembered is that in some patients, in spite of drug withdrawal and high steroid therapy, a relapsing and chronic course might ensue [22].

Since genetic factors are involved in the pathogenesis of **DHS**, relatives should be instructed about **DHS **and their enhanced risk of developing similar adverse reactions should they take dapsone [10].

## Conclusion

A high index of suspicion is needed for early diagnosis of **DHS**. Patients initiated on dapsone for various indications need to be observed carefully for the development of the **DHS**. If and when this occurs, **DHS **can be mistaken for progression of the primary disease. If the drug is not withdrawn, it could have deleterious and potentially fatal effects due to major organ dysfunction. In our patient, the association of hypoxemic pulmonary disease, pleural effusions and anemia led to serious problems with oxygenation. The prompt withdrawal of the offending drug, administration of glucocorticoids and supportive management led to rapid recovery in our patient

## References

[B1] Zhu YI, Stiller MJ (2001). Dapsone and sulfones in dermatology: overview and update. J Am Acad Dermatol.

[B2] Singletary EM, Rochman AS, Bodmer JC, Holstege CP (2005). Envenomations
1. Med Clin North Am.

[B3] Leslie KS, Gaffney K, Ross CN, Ridley S, Barker TH, Garioch JJ (2003). A near fatal case of the dapsone hypersensitivity syndrome in a patient with urticarial vasculitis. Clin Exp Dermatol.

[B4] Pavithran K, Bindu V (1999). Dapsone syndrome: hepatitis-B infection a risk factor for its development?. Int J Lepr Other Mycobact Dis.

[B5] Knowles SR, Shapiro LE, Shear NH (2003). Reactive metabolites and adverse drug reactions: clinical considerations. Clin Rev Allergy Immunol.

[B6] Rao PN, Lakshmi TS (2001). Increase in the incidence of dapsone hypersensitivity syndrome--an appraisal. Lepr Rev.

[B7] Christiansen J, Tegner E, Irestedt M (1999). Dapsone hypersensitivity syndrome in a patient with cutaneous lupus erythematosus. Acta Derm Venereol.

[B8] Reeve PA, Ala J, Hall JJ (1992). Dapsone syndrome in Vanuatu: a high incidence during multidrug treatment (MDT) of leprosy. J Trop Med Hyg.

[B9] Itha S, Kumar A, Dhingra S, Choudhuri G (2003). Dapsone induced cholangitis as a part of dapsone syndrome: a case report. BMC Gastroenterol.

[B10] Camus P, Bonniaud P, Fanton A, Camus C, Baudaun N, Foucher P (2004). Drug-induced and iatrogenic infiltrative lung disease
8. Clin Chest Med.

[B11] Begbie S, Burgess KR (1993). Maloprim-induced pulmonary eosinophilia. Chest.

[B12] Janier M, Guillevin L, Badillet G (1994). Pulmonary eosinophilia associated with dapsone. Lancet.

[B13] Arunthathi S, Raju S (1998). Dapsone induced pulmonary eosinophilia without cutaneous allergic manifestations--an unusual encounter--a case report. Acta Leprol.

[B14] Jaffuel D, Lebel B, Hillaire-Buys D, Pene J, Godard P, Michel FB, Blayac JP, Bousquet J, Demolyi P (1998). Eosinophilic pneumonia induced by dapsone. BMJ.

[B15] Davidson AC, Bateman C, Shovlin C, Marrinan M, Burton GH, Cameron IR (1988). Pulmonary toxicity of malaria prophylaxis. BMJ.

[B16] Tobin-D'Angelo MJ, Hoteit MA, Brown KV, Ray SM, King MD (2004). Dapsone-induced hypersensitivity pneumonitis mimicking Pneumocystis carinii pneumonia in a patient with AIDS. Am J Med Sci.

[B17] Corp CC, Ghishan FK (1998). The sulfone syndrome complicated by pancreatitis and pleural effusion in an adolescent receiving dapsone for treatment of acne vulgaris. J Pediatr Gastroenterol Nutr.

[B18] Mery L, Dega H, Prost C, Dubertret L (2003). [Dapsone-induced sensory peripheral neuropathy]. Ann Dermatol Venereol.

[B19] Fine JD, Katz SI, Donahue MJ, Hendricks AA (1983). Psychiatric reaction to dapsone and sulfapyridine
1. J Am Acad Dermatol.

[B20] Hoffbrand BI (1978). Dapsone and renal papillary necrosis
1. Br Med J.

[B21] Volcheck GW (2004). Clinical evaluation and management of drug hypersensitivity
5. Immunol Allergy Clin North Am.

[B22] McKenna JK, Leiferman KM (2004). Dermatologic drug reactions
2. Immunol Allergy Clin North Am.

[B23] McKenna KE, Robinson J (1997). The dapsone hypersensitivity syndrome occurring in a patient with dermatitis herpetiformis. Br J Dermatol.

[B24] Prussick R, Shear NH (1996). Dapsone hypersensitivity syndrome. J Am Acad Dermatol.

[B25] Krishnaswamy G (2001). Treatment strategies for bronchial asthma: an update
33. Hosp Pract (Off Ed).

[B26] Shakoory B, Fitzgerald SM, Lee SA, Chi DS, Krishnaswamy G (2004). The role of human mast cell-derived cytokines in eosinophil biology
14. J Interferon Cytokine Res.

[B27] Rhen T, Cidlowski JA (2005). Antiinflammatory action of glucocorticoids - New mechanisms for old drugs
1. New England Journal of Medicine.

[B28] Fitzgerald SM, Chi DS, Hall HK, Reynolds SA, Aramide O, Lee SA, Krishnaswamy G (2003). GM-CSF induction in human lung fibroblasts by IL-1beta, TNF-alpha, and macrophage contact
24. J Interferon Cytokine Res.

[B29] Chogle A, Nagral A, Soni A, Agale S, Jamadar Z (2000). Dapsone hypersensitivity syndrome with coexisting acute hepatitis E. Indian J Gastroenterol.

[B30] Gupta A, Eggo MC, Uetrecht JP, Cribb AE, Daneman D, Rieder MJ, Shear NH, Cannon M, Spielberg SP (1992). Drug-induced hypothyroidism: the thyroid as a target organ in hypersensitivity reactions to anticonvulsants and sulfonamides
1. Clin Pharmacol Ther.

